# Site specific incidence rate of virulence related genes of enteroaggregative *Escherichia coli* and association with enteric inflammation and growth in children

**DOI:** 10.1038/s41598-021-02626-z

**Published:** 2021-11-30

**Authors:** Rina Das, Parag Palit, Md. Ahshanul Haque, Mustafa Mahfuz, A. S. G. Faruque, Tahmeed Ahmed

**Affiliations:** Nutrition and Clinical Services Division, icddr,b, 68 Shaheed Tajuddin Ahmed Sharani, Dhaka, 1212 Bangladesh

**Keywords:** Biomarkers, Gastroenterology, Medical research, Risk factors

## Abstract

There is a lack of information highlighting the possible association between strain carrying genes of enteroaggregative *Escherichia coli* (EAEC) and environmental enteric dysfunction (EED) and on linear growth during childhood. Strain carrying genes of EAEC from stool samples collected from 1705 children enrolled in the MAL-ED birth cohort were detected by TaqMan Array Cards. We measured site-specific incidence rate by using Poisson regression models, identified the risk factors and estimated the associations of strain carrying genes of EAEC with the composite EED score and linear growth at 24 months of age. Overall highest incidence rate (43.3%) was found among children having infection with the *aggR* gene, which was the greatest in Tanzania (56.7%). Low maternal education, lack of improved floor, and ownership of domestic cattle were found to be risk factors for EAEC infection. In the multivariate models, after adjusting the potential covariates, strain carrying genes of EAEC showed strong positive associations with the EED scores and with poor linear growth at 24 months of age. Our analyses may lay the cornerstone for a prospective epidemiologic investigation for a potential vaccine development aimed at reducing the burden of EAEC infections and combat childhood malnutrition.

## Introduction

Childhood malnutrition is strongly associated with the risk of death from diarrhea, pneumonia, and other infectious diseases and is associated with growth failure, cognitive delay, and loss of productivity^1^. The main bacterial enteric pathogen in less-developed countries, particularly among children aged 2 to 24 months, is *Escherichia coli*^2, 3^. Among the major categories of diarrhoeagenic *E. coli*, enteroaggregative *E. coli* (EAEC) constitute a significant health risk and remains an important cause of infant mortality in developing countries^4^.

Evidence suggests that gastrointestinal infections with several enteropathogens, including EAEC, are linked with childhood malnutrition^5–7^. EAEC has been increasingly recognized as important enteropathogens since their initial discovery by patterns of adherence to HEp-2 (Human epithelial type 2) cells in *E. coli* isolates from Chilean children with diarrhea^8^. The genetic determinants and biological mechanisms for the virulence of EAEC have been reported to be mediated by a complex array of interacting traits that reside on both the chromosome and the plasmid^9^. As presently defined, EAEC is heterogeneous with regards to their genetic content^10^.

The aagR plasmid, harbored by most EAEC strains encodes a transcriptional activator of the AtaC/XylS class known as AggR^11^, which in turn, regulates the expression of virulence proteins involved in the adherence to mucosal secretions and thereby promoting intestinal colonization^12, 13^. AggR also activates the expression of the genes encoding dispersin (aap), the dispersin translocator Aat, the Aai chromosomal type IV secretion system (aaiA-Y), as well as the plasmid-borne *aatA* gene, which encodes an ABC (ATP-binding cassette) transporter^14^. Consequently, the aar gene of EAEC encodes a small protein named Aar (AggR activated regulator), whose expression is activated by AggR, and serves as a negative regulator of AggR^15^. Henceforth, there is a complex interplay between AggR and Aar, in relation to the virulence of EAEC and its consequent pathogenesis^16^.

Environmental Enteric Dysfunction (EED) is a subclinical intestinal disorder that is highly prevalent in low- and middle-income countries (LMICs)^17^ and is attributable to the infection by many environmental enteropathogens of bacterial, viral, and parasitic nature^18, 19^. Mechanisms contributing to growth failure in EED include intestinal leakiness and elevated gut permeability, gut inflammation, bacterial translocation, nutrient malabsorption, and systemic inflammation^17^. However, the definite contribution of different strains of this EAEC with linear growth faltering and EED remains poorly understood^20^.

The main goal of our study was to estimate the site-specific incidence rates of potential strain carrying genes of EAEC and their possible associations with the composite EED score and the consequent growth failure among children at 24 months of age.

## Results

### General characteristics of the study population and incidence rate of strain carrying genes of EAEC

A total of 34,622 monthly stool samples were collected from 1715 participants who completed the follow-up to 24 months. All the stool samples collected over this time from all the participants at the different study sites were assessed for the presence of strain carrying genes associated with EAEC using TaqMan Array Cards (TAC). The general characteristics of the study children are presented in Table 1.

**Table 1:** General characteristics of the MAL-ED study Children (n = 1715) from Bangladesh, India, Nepal, Pakistan, South Africa, Tanzania, Brazil, and Peru from November 2009 to February 2012.

Characteristics n (%)	Bangladesh	Brazil	India	Nepal	Peru	Pakistan	South Africa	Tanzania	Overall
Gender (Male)	108 (51.43)	89 (53.94)	105 (46.26)	122 (53.74)	105 (54.12)	120 (48.78)	120 (50.63)	105 (50.24)	874 (50.96)
Days of exclusive breastfeeding^a^	143.2 ± 42.7	93.7 ± 57.8	105.4 ± 42.9	92.5 ± 54.5	89.5 ± 61.3	19.9 ± 22.7	38.6 ± 26.3	62.2 ± 35	78.6 ± 57.7
Birth weight (Kg)^a^	2.8 ± 0.4	3.4 ± 0.5	2.9 ± 0.4	3.0 ± 0.4	3.1 ± 0.4	2.7 ± 0.4	3.2 ± 0.5	3.2 ± 0.5	3.0 ± 0.5
Weight for age z score at enrollment^a^	− 1.3 ± 0.9	− 0.2 ± 1.0	− 1.3 ± 1.0	− 0.9 ± 1.0	− 0.6 ± 0.9	− 1.4 ± 1.0	− 0.4 ± 1.0	− 0.1 ± 1.1	− 0.8 ± 1.1
Length for age z score at enrollment^a^	− 1.0 ± 1.0	− 0.8 ± 1.1	− 1.0 ± 1.1	− 0.7 ± 1.0	− 0.9 ± 1.0	− 1.3 ± 1.1	− 0.7 ± 1.0	− 1.0 ± 1.1	− 0.9 ± 1.1
Length for age z score at 24 months^a^	− 2.0 ± 0.9	0.0 ± 1.1	− 1.9 ± 1.0	− 1.3 ± 0.9	− 1.9 ± 0.9	–	− 1.7 ± 1.1	− 2.7 ± 1.0	− 1.7 ± 1.2
Weight for length z score at 24 months^a^	− 0.8 ± 0.9	0.5 ± 1.4	− 0.9 ± 0.9	− 0.3 ± 0.9	0.3 ± 0.9	–	0.5 ± 1.0	0.1 ± 1.0	− 0.1 ± 1.1
Maternal age (months)^a^	25 ± 5	25.4 ± 5.6	23.9 ± 4.2	26.6 ± 3.7	24.8 ± 6.3	28.1 ± 5.9	27 ± 7.2	29.1 ± 6.5	26.3 ± 5.9
Maternal BMI^a,b^	22.3 ± 3.4	25.7 ± 4.4	22.0 ± 4.0	25.1 ± 3.2	24.9 ± 3.7	21.5 ± 3.8	27 ± 5.5	22.9 ± 3.2	23.9 ± 4.4
Maternal education level (< 6y)	133 (63.3)	22 (13.3)	80 (35.2)	59 (26)	44 (22.7)	202 (82.1)	5 (2.1)	75 (35.9)	620 (36.2)
Mother has < 3 living children	160 (76.2)	113 (68.5)	157 (69.8)	199 (87.7)	111 (57.2)	105 (42.7)	141 (59.5)	58 (27.8)	1044 (61)
Ownership of cattle	1 (0.5)	0	5 (2.2)	3 (1.3)	0	146 (59.4)	33 (13.9)	157 (75.1)	345 (20.1)
Ownership of chicken	3 (1.4)	1 (0.6)	14 (6.2)	73 (32.2)	75 (38.7)	144 (62.3)	87 (37.2)	204 (97.6)	601 (35.4)
Routine treatment of drinking water	130 (61.9)	10 (6.1)	7 (3.1)	98 (43.2)	32 (16.5)	0	12 (5.1)	12 (5.7)	301 (17.6)
Improved drinking water source	210 (100)	165 (100)	227 (100)	227 (100)	184 (94.9)	246 (100)	196 (82.7)	89 (42.6)	1544 (90.0)
Improved floor	204 (97.1)	165 (100)	222 (97.8)	109 (48)	69 (35.6)	81 (32.9)	231 (97.5)	13 (6.2)	1094 (63.8)
Improved sanitary latrine	210 (100)	165 (100)	121 (53.3)	227 (100)	66 (34)	197 (80.1)	232 (97.9)	19 (9.1)	1237 (72.1)
Monthly income < 150 USD	69 (32.9)	161 (97.6)	19 (8.4)	106 (46.7)	58 (29.9)	115 (46.8)	179 (75.5)	0	707 (41.2)
< 2 people live in per room	8 (3.8)	141 (85.5)	46 (20.3)	126 (55.5)	122 (62.9)	27 (10.9)	201 (84.8)	95 (45.5)	766 (44.7)
Average serum zinc level (mmol/l)^c,d^	11.3 (10.6, 12.1)	14 (13, 14.9)	9.1 (8.6, 9.6)	11.2 (10.4, 12.2)	14.8 (13.1, 17.9)	8.9 (7.7, 10)	22.9 (14.3, 32.9)	11.1 (9.9, 12.3)	11.3 (9.6, 13.7)
Average AGP (mg/dL)^c,d^	84.3 (71.5,105.3)	95.7 (81, 117)	97 (83, 110)	117.7 (102.7, 139)	115 (98, 130.3)	93 (77.5, 111.8)	126 (107.3, 153, 7)	114.3 (97.7, 138.7)	106.3 (87, 127)

^a^Mean ± Standard deviation.

^b^BMI: body mass index;

^c^Median (IQR).

^d^Average of 7, 15 and 24-months serum zinc and plasma AGP (alpha-1-acid glycoprotein) level.

The incidence rates of the strain carrying genes of EAEC in the stool samples collected across all the 8 study sites over the 24 months’ study period have been shown in Table 2. The overall incidence rate of *aggR* was highest (43.3%). The incidence of strain carrying gene *Aar* associated with EAEC was highest in Tanzania (58.1%). It was also observed that the overall incidence of both *aaiC* and *aatA* strain carrying gene was lowest among all the sites.

**Table 2:** The incidence rate of strain carrying genes of EAEC infection across each of the eight study sites (Bangladesh, India, Nepal, Pakistan, South Africa, Tanzania, Brazil, and Peru) from November 2009 to February 2012.

Sites	EAEC-*aaiC*	EAEC-A*ar*	EAEC-*aatA*	EAEC-*aggR*	EAEC-aaiC and aatA
	Incidence rate (95% CI)				
Bangladesh	20.7 (19.4, 22.1)	50.6 (48.6, 52.8)	32.0 (30.4, 33.7)	51.6 (49.5, 53.8)	8.9 (8.7, 9.1)
Brazil	20.4 (20.7, 24.2)	22.3 (20.6, 24.1)	22.4 (20.7, 24.2)	21.7 (20.0, 23.4)	13.2 (12.9, 13.5)
India	35.4 (33.8, 37.1)	51.8 (49.8. 53.9)	52.9 (50.9, 55.0)	54.5 (52.4, 56.6)	23.7 (23.4, 24.0)
Nepal	31.2 (29.7, 32.8)	38.4 (36.7, 40.2)	38.2 (36.5, 39.9)	39.9 (38.1, 41.7)	19.7 (19.5, 19.9)
Peru	34.1 (32.4, 35.9)	51.6 (49.5, 53.8)	51.9 (49.8, 54.1)	49.8 (47.7, 51.9)	23.7 (23.4, 24.0)
Pakistan	15.6 (14.5, 16.7)	30.3 (28.7, 32.0)	30.3 (28.8, 32.0)	33.0 (31.3, 34.7)	9.1 (8.9, 9.3)
South Africa	23.5 (22.1, 24.9)	32.1 (30.5, 33.7)	28.7 (27.2, 30.2)	32.7 (31.1, 34.4)	14.8 (14.6, 15.0)
Tanzania	33.5 (31.8, 27.8)	58.1 (55.9, 60.5)	56.3 (54.1, 58.6)	56.7 (54.5, 59.3)	24.1 (23.8, 24.4)
Overall	27.3 (26.7, 27.8)	42.6 (41.9, 43.3)	39.7 (39.0, 40.4)	43.3 (42.6, 44.2)	17.3 (17.2, 17.4)

*95% CI*: 95% confidence interval; Incidence rates were calculated using Poisson regression where outcome variables were a number of infections of EAEC ( strain carrying genes) and offset variables were log of the number of follow up.

### Factors associated with strain carrying genes of EAEC

Factors associated with strain carrying genes associated with EAEC across all study sites were identified using Poisson regression (Table 3). The incidence rate for infection of EAEC in female children was comparable with male children. Additionally, household ownership of cattle among children with infections by the genomic strains of *aaiC* [IRR: 0.90 (95% CI: 0.83, 0.97); *p* = 0.010], *Aar* [IRR: 0.94 (95% CI: 0.88, 1.0); *p* = 0.042], and *aggR* [IRR: 0.94 (95% CI: 0.88, 1.0); *p* = 0.048]; improved floor among *aaiC* strain infected children [IRR: 0.93 (95% CI: 0.87, 0.99; *p* = 0.029]; monthly income less than 150 USD among children infected with the genomic strains of *Aar* and *aggR*; maternal education in years among children infected with *aaiC* [IRR: 0.99 (95% CI: 0.99, 1.0); *p* = 0.030] and *Aar* [IRR: 0.99 (95% CI: 0.99, 1.0); *p* = 0.006] genomic strains were associated and found to be statistically significant.

**Table 3:** Factors associated with strain carrying genes of EAEC detection in monthly surveillance stool samples across each of the eight study sites (Bangladesh, India, Nepal, Pakistan, South Africa, Tanzania, Brazil, and Peru) from November 2009 to February 2012.

Factors	EAEC-*aaiC*		EAEC-A*ar*		EAEC-*aatA*		EAEC-*aggR*		EAEC-*aaiC and aatA*	
	IRR (95% CI)	*P-value*	IRR (95% CI)	*P-value*	IRR (95% CI)	*P-value*	IRR (95% CI)	*P-value*	IRR (95% CI)	*P-value*
**Sex**										
Female	Reference									
Male	1.0 (0.96, 1.04)	0.920	1.02 (0.98, 1.05)	0.293	1.01 (0.98, 1.05)	0.397	1.02 (0.99, 1.05)	0.287	0.98 (0.97, 0.99)	< 0.001
**Duration of exclusive breastfeeding (months)**	0.99 (0.97–1.00)	0.057	1.0 (0.99, 1.01)	0.565	1.0 (0.98, 1.01)	0.440	1.00 (0.99, 1.01)	0.865	0.99 (0.98, 0.99)	< 0.001
**Household ownership**										
Cattle	0.90 (0.83, 0.97)	0.010	0.94 (0.88, 1.0)	0.042	0.98 (0.91, 1.04)	0.456	0.94 (0.88, 1.0)	0.048	0.92 (0.90, 0.94)	< 0.001
Chicken	1.03 (0.97, 1.09)	0.329	0.97 (0.92, 1.01)	0.166	0.96 (0.92, 1.01)	0.115	0.98 (0.94, 1.03)	0.454	1.03 (1.01, 1.04)	0.001
**Enrollment WAZ**^a^	1.0 (0.97, 1.02)	0.687	1.0 (0.98, 1.02)	0.900	0.99 (0.97, 1.01)	0.270	0.99 (0.97,1.01)	0.260	0.99 (0.98, 0.99)	0.001
**Improved floor**										
No	Reference									
Yes	0.93 (0.87, 0.99)	0.029	0.98 (0.93, 1.03)	0.465	0.97 (0.92, 1.02)	0.279	0.96 0.91, 1.01)	0.160	0.90 (0.88, 0.91)	< 0.001
**Improved drinking water source**										
No	Reference									
Yes	0.97 (0.89, 1.05)	0.412	1.03 (0.96,1.10)	0.379	1.02 (0.95, 1.09)	0.615	1.02 (0.95, 1.09)	0.624	1.00 (0.98, 1.03)	0.743
**Routine treatment of drinking water**										
No	Reference									
Yes	0.97 (0.89, 1.05)	0.412	1.01 (0.96, 1.06)	0.824	1.02 (0.97, 1.08)	0.377	1.01 (0.96, 1.06)	0.815	1.03 (1.01, 1.05)	< 0.001
**Improved sanitation**										
No	Reference									
Yes	1.05 (0.98, 1.12)	0.156	0.98 (0.93, 1.03)	0.497	0.98 (0.93, 1.03)	0.503	0.98 (0.93, 1.03)	0.350	1.03 (1.01, 1.04)	0.004
**Maternal age (years)**	1.0 (0.99–1.0)	0.282	1.0 (1.0, 1.0)	0.478	1.00 (1.0, 1.0)	0.906	1.00 (1.0, 1.0)	0.905	0.99 (0.99–1.00)	0.189
**Mother has < 3 living children**										
No	Reference									
Yes	0.96 (0.91, 1.02)	0.214	1.0 (0.95, 1.04)	0.860	1.0 (0.95, 1.04)	0.908	1.00 (0.95, 1.04)	0.869	0.97 (0.95, 0.99)	< 0.001
** < 2 people live in per room**										
No	Reference									
Yes	1.0 (0.95, 1.05)	0.864	0.99 (0.95, 1.03)	0.563	0.98 (0.94, 1.02)	0.393	0.98 (0.94, 1.02)	0.256	0.98 (0.96, 0.99)	< 0.001
**Monthly income < 150 USD**										
No	Reference									
Yes	0.99 (0.94, 1.04)	0.805	0.96 (0.92, 1.0)	0.056	0.96 (0.92, 1.0)	0.079	0.96 (0.92, 1.0)	0.053	1.00 (0.99, 1.02)	0.070
**Maternal education (years)**	0.99 (0.99, 1.0)	0.030	0.99 (0.99, 1.0)	0.006	1.0 (0.99, 1.0)	0.456	1.00 (0.99, 1.0)	0.122	0.99 (0.98, 0.99	< 0.001
**Country**										
Bangladesh	Reference									
Brazil	1.09 (0.96, 1.24)	0.173	0.48 (0.43, 0.53)	< 0.001	0.75 (0.67, 0.84)	< 0.001	0.46 (0.41, 0.51)	< 0.001	1.60 (1.54, 1.66)	< 0.001
India	1.71 (1.55, 1.89)	< 0.001	1.03 (0.96, 1.11)	0.384	1.66 (1.53, 1.79)	< 0.001	1.06 (0.98, 1.14)	< 0.001	2.76 (2.68, 2.85)	< 0.001
Nepal	1.46 (1.32, 1.61)	< 0.001	0.80 (0.74, 0.86)	< 0.001	1.22 (1.12, 1.32)	< 0.001	0.79 (0.74, 0.86)	< 0.001	2.17 (2.10, 2.23)	< 0.001
Peru	1.58 (1.41, 1.77)	< 0.001	1.05 (0.96, 1.14)	0.314	1.64 (1.49, 1.80)	< 0.001	0.97 (0.89, 1.06)	0.453	2.60 (2.51, 2.68)	< 0.001
Pakistan	0.69 (0.61, 0.79)	< 0.001	0.64 (0.58, 0.71)	< 0.001	0.97 (0.87, 1.07)	0.539	0.66 (0.60, 0.73)	< 0.001	0.96 (0.92, 0.99)	0.002
South Africa	1.13 (0.99, 1.27)	0.061	0.71 (0.65, 0.79)	< 0.001	0.97 (0.87, 1.07)	0.505	0.70 (0.64, 0.77)	< 0.001	1.76 (1.70, 1.83)	< 0.001
Tanzania	1.55 (1.34, 1.80)	< 0.001	1.24 (1.10, 1.39)	< 0.001	1.81 (1.61, 2.05)	< 0.001	1.13 (1.01, 1.27)	0.032	2.62 (2.52, 2.74)	< 0.001

*Model* Poisson regression, *IRR* incidence rate ratio; ^a^WAZ: Weight-for-age z score.

Dependent variable: Number of infections during follow up (1–24 m); Offset: Log of the total number of follow up; alpha (α): dispersion parameter; Adjusted for the site and all variables included in the multivariable model.

The incidence rate of *aaiC* and *aatA* positive strains was higher for the sites of India, Nepal, Peru, and Tanzania, while that of the genomic strain of *Aar* was the greatest in Tanzania. Consequently, the incidence rate of *aatA* genomic strain was the lowest in Brazil and that of *aggR* was the lowest in Nepal, South Africa, Brazil, and Pakistan. For concomitant infection by the positive strains of *aaiC* and *aatA*, the incidence rate was greater in India, Nepal, Peru, Tanzania compared to the Bangladesh site (Table 3).

### Association between the strain carrying genes of EAEC and child growth

Infections with the strain carrying genes of EAEC, namely: *Aar, aatA, aggR,* and the concomitant presence of *aaiC* and *aatA* strains were associated with poor linear growth (difference in 24 months LAZ: length-for-age z score), with a stronger association being observed for all the study sites (Table 4). In Bangladesh, *aaiC* [− 1.32 difference in 24 months LAZ (95% CI: − 2.48, − 0.16); *p* < 0.027] and both *aaiC* and *aatA* carrying genes [− 1.36 difference in 24 months LAZ (95% CI: − 1.73, − 0.99; *p* < 0.001] had a negative association with LAZ. In Peru, *Aar* strain [− 0.96 difference in 24 months LAZ (95% CI: − 1.84, − 0.09); *p* = 0.030] was negatively associated with LAZ. In South Africa *aatA* [− 1.02 difference in 24 months LAZ (95% CI: − 2.00, − 0.05); *p* = 0.040]; in Tanzania, *aatA* [− 2.04 difference in 24 months LAZ (95% CI: − 3.55, − 0.53); *p* = 0.009] was negatively associated with LAZ. For the other four countries (South Africa, Brazil, Nepal and India), strain carrying genes associated with EAEC were negatively associated with LAZ but were not statistically significant.

**Table 4:** Strain carrying genes of EAEC and burden on child growth at 24 months across each of the study sites (except Pakistan) from November 2009 to February 2012.

Sites	EAEC*-aaiC*		EAEC*-Aar*		EAEC-*aatA*		EAEC*-aggR*		EAEC*-aaiC* and *aatA*	
	Coef. (95% CI)	*P-value*	Coef. (95% CI)	*P-value*	Coef. (95% CI)	*P-value*	Coef. (95% CI)	*P-value*	Coef. (95% CI)	*P-value*
**Length-for-age z score**										
Bangladesh	− 1.32 (− 2.48, − 0.16)	0.027	− 0.59 (− 1.50, 0.32)	0.205	− 0.19 (− 1.07, 0.70)	0.679	− 0.35 (− 1.17, 0.46)	0.392	− 1.36 (− 1.73, − 0.99)	< 0.001
Brazil	− 0.49 (− 1.91, 0.93)	0.493	− 0.77 (− 2.09, 0.54)	0.248	− 0.83 (− 2.08, 0.42)	0.190	− 1.16 (− 2.44, 0.11)	0.074	− 0.62 (− 1.03, − 0.21)	0.003
India	0.24 (− 0.74, 1.23)	0.624	− 0.85 (− 1.72, 0.01)	0.054	− 0.78 (− 1.70, 0.14)	0.095	− 0.79 (− 1.68, 0.10)	0.081	− 0.49 (− 0.74, − 0.25)	< 0.001
Nepal	0.78 (− 0.20, 1.76)	0.116	0.42 (− 0.38, 1.23)	0.300	0.26 (− 0.60, 1.12)	0.550	0.32 (− 0.47, 1.12)	0.423	0.65 (0.41, 0.89)	< 0.001
Peru	− 0.55 (− 1.52, 0.42)	0.261	− 0.96 (− 1.84, − 0.09)	0.030	− 0.48 (− 1.40, 0.44)	0.303	− 0.58 (− 1.46, 0.31)	0.203	− 0.73 (− 0.96, − 0.49)	< 0.001
South Africa	− 0.93 (− 2.23, 0.37)	0.160	− 0.93 (− 1.96, 0.10)	0.078	− 1.02 (− 2.00, − 0.05)	0.040	− 0.65 (− 1.70, 0.41)	0.228	− 0.57 (− 0.92, − 0.22)	0.001
Tanzania	0.70 (− 1.25, 2.66)	0.476	− 0.55 (− 2.44, 1.33)	0.561	− 2.04 (− 3.55, − 0.53)	0.009	− 0.64 (− 2.34, 1.05)	0.453	0.18 (− 0.22, 0.58)	0.390
Overall	− 0.19 (− 0.62, 0.24)	0.391	− 0.57 (− 0.94, − 0.20)	0.003	0.60 (− 0.97, − 0.22)	0.002	− 0.51 (− 0.88, − 0.15)	0.006	− 0.29 (− 0.41, − 0.18)	< 0.001

Adjusted in the linear regression model for sex, birth weight, exclusive breastfeeding, WAMI Index (water/sanitation, assets, maternal education, and income); enrollment length-for-age z score; maternal BMI; poultry and cattle in house, a mother has less than 3 living children, average serum zinc level, average AGP (alpha-1-acid glycoprotein) level, presence of co-pathogens (*Campylobacter*, LT-ETEC, ST-ETEC, *Shigella*/EIEC, and *Giardia*) and site for overall estimate.

Coef.: Coefficient; CI: Confidence interval.

Dependent variable: length-for-age z score at 24 m; Independent variables: the burden of strain carrying genes associated with EAEC.

### Association between strain carrying genes associated with EAEC and enteric inflammation

After adjusting for the potential covariates like age, sex, WAMI index (water/sanitation, assets, maternal education, and income); enrollment length-for-age z score; maternal BMI; the number of children in the household, presence of poultry/cattle in the household, seasonality, serum zinc level, AGP (alpha-1-acid glycoprotein), presence of co-pathogens (*Campylobacter*, LT-ETEC, ST-ETEC, *Shigella*/EIEC, and *Giardia*), site for the overall estimate and age as the time variable in GEE model for strain carrying genes of EAEC infection were also clearly and consistently associated with increased EED score (Table 5) across all sites except Pakistan and Brazil. In case of the overall effect, the presence of all the strain carrying genes was associated with the EED score except *aaiC.* The same findings were observed in India, Nepal and Peru individually except *aaiC* strain carrying gene. In Pakistan and Brazil, there was no statistically significant relationship found among all the strain carrying genes. In Tanzania, only *aaiC* gene was non-significantly associated with the EED score. *Aar*, and *aggR*, strain carrying genes had a positive relationship with EED score among the children from Bangladesh. In South Africa except for *Aar* and *aaiC*, all other genes were significantly associated with EED score (Table 5). In Nepal and Peru both *aaiC* and *aatA* strain carrying genes had a statistically significant positive relationship with EED score.

**Table 5:** Association between strain carrying genes of EAEC and EED score across each of the eight study sites (Bangladesh, India, Nepal, Pakistan, South Africa, Tanzania, Brazil, and Peru) from November 2009 to February 2012.

Site	EAEC*-aaiC*		EAEC*-Aar*		EAEC-*aatA*		EAEC*-aggR*		EAEC*-aaiC* and *aatA*	
	Coef. (95% CI)	*P-value*	Coef. (95% CI)	*P-value*	Coef. (95% CI)	*P-value*	Coef. (95% CI)	*P-value*	Coef. (95%CI)	*P-value*
**EED score**										
Bangladesh	0.10 (− 0.15, 0.35)	0.430	0.31 (0.12, 0.51)	0.002	0.15 (− 0.06, 0.36)	0.159	0.47 (0.27, 0.67)	< 0.001	− 0.11 (− 0.44, 0.22)	0.501
Brazil	− 0.05 (− 0.39, 0.30)	0.790	0.01 (− 0.32, 0.34)	0.943	0.28 (− 0.05, 0.62)	0.100	0.29 (− 0.06, 0.64)	0.101	0.12 (− 0.29, 0.54)	0.570
India	0.05 (− 0.14, 0.24)	0.594	0.50 (0.32, 0.68)	< 0.001	0.45 (0.27, 0.63)	< 0.001	0.48 (0.30, 0.67)	< 0.001	0.16 (− 0.05, 0.36)	0.139
Nepal	0.17 (− 0.03, 0.37)	0.090	0.46 (0.27, 0.65)	< 0.001	0.47 (0.29, 0.66)	< 0.001	0.47 (0.28, 0.66)	< 0.001	0.28 (0.08, 0.51)	0.014
Peru	0.17 (− 0.08, 0.43)	0.177	0.62 (0.39, 0.85)	< 0.001	0.64 (0.41, 0.87)	< 0.001	0.60 (0.37, 0.82)	< 0.001	0.28 (0.0002, 0.56)	0.050
Pakistan	− 0.02 (− 0.27, 0.24)	0.898	0.15 (− 0.05, 0.35)	0.147	0.17 (− 0.03, 0.36)	0.096	0.15 (− 0.05, 0.35)	0.133	0.08 (− 0.23, 0.39)	0.596
South Africa	0.11 (− 0.17, 0.39)	0.455	0.23 (− 0.01, 0.48)	0.064	0.26 (0.01, 0.51)	0.044	0.32 (0.08, 0.56)	0.010	0.04 (− 0.29, 0.37)	0.809
Tanzania	0.02 (− 0.22, 0.26)	0.861	0.46 (0.23, 0.70)	< 0.001	0.55 (0.32, 0.78)	< 0.001	0.50 (0.26, 0.74)	< 0.001	0.18 (− 0.08, 0.44)	0.176
Overall	0.08 (− 0.01, 0.16)	0.076	0.42 (0.34, 0.49)	< 0.001	0.42 (0.34, 0.49)	< 0.001	0.45 (0.38, 0.53)	< 0.001	0.16 (0.06, 0.25)	0.002

Adjusted in GEE model for sex, age, WAMI Index (water/sanitation, assets, maternal education, and income); enrollment length-for-age z score; maternal BMI; the number of children, poultry/cattle in house, seasonality, serum zinc level, AGP (alpha-1-acid glycoprotein), presence of co-pathogens (*Campylobacter*, LT-ETEC, ST-ETEC, *Shigella*/EIEC, and *Giardia*), site for the overall estimate, and age as the time variable. EED score (AAT = alpha-1-anti-trypsin; MPO = myeloperoxidase; NEO = neopterin) Coef.: Coefficient; CI: Confidence interval The dependent variable was log (MPO, NEO, and AAT); Independent variables: the presence of strain carrying genes associated with EAEC at each month.

## Discussion

To our knowledge, this is the first study investigating the association between different strain carrying genes of EAEC with linear growth and enteric inflammation in children from birth until 2 years of age. Several EAEC strain carrying genes have been used in case–control and epidemiological studies in recent years, with *aatA, aap, aagR, astA,* and *aafA* being among the most common for EAEC diagnosis^21–23^.

We documented an overall high incidence rate of the concomitant presence of *aggR* gene carrying strains of EAEC across all eight study sites. The different clinical and nutritional outcomes associated with EAEC infection included: poor child growth and development during the study period and changes in the status of intestinal inflammation and provide stern challenges for understanding pathobiology and proposing potential therapeutic approaches for EAEC^8^. In this scenario, EAEC strain heterogeneity is a key contributor to these effects, but very little is known about the individual genomic strains responsible for this^8^.

In our study, the overall incidence rate for the genomic strain of *aaiC* was the lowest across all the study sites. Previously, an incidence rate of 38% for the *aaiC* gene among children under 5 years of age was reported in the Global Enteric Multicenter Study (GEMS)^10^. The incidence of *aaiC, aggR, aatA, aaR,* and the concomitant presence of *aaiC* and *aatA* strain carrying genes were higher among the study participants in Tanzania. Similar findings were observed in the case of traveler’s diarrhea in Guatemala and Mexico^24^. Many plasmid-encoded *(AAFs, AggR* itself*, Aap,* and *Aat)* and chromosomal-encoded *(pheU* pathogenicity island*)* virulence determinants are regulated *by aggR*^15^. Reduced viability of infected *G. mellonella* larvae with *an aggR* mutant strain and elevated virulence of atypical EAEC strains were discovered in a study, suggesting that EAEC virulence is linked to the *AggR* regulon^25^.

The low incidence of the *aaiC* gene in our study compared to the other strain carrying genes of EAEC may be additional support for the plasmid-mediated gene transfer of the pAA. In several studies, the authors suggested that *aggR* was not a feasible virulence marker for the diagnosis of EAEC infections since this gene alone was not a considerably sensitive target in comparison to the *aatA* gene^26, 27^. Our study findings regarding *aaiC* being an incongruent marker for EAEC identification were found to be consistent with a study conducted in southern Mozambique^23^.

The association between strain carrying genes associated with EAEC and malnutrition is not entirely clear, although plausible models for pathogenesis can be proposed. The *Aar* gene, which has been hypothesized to act directly or indirectly as a virulence suppressor, was present among the children in Tanzania^28^. Similar findings have also been reported in Mali and Brazil and these findings may strongly support a significant role for the *Aar* gene in the epidemiology of EAEC infection^23^. We also observed that there was poor linear growth among the children from Peru who were infected with the genomic strain of *Aar*; which may be pointed towards increased pathogenicity of *Aar* gene.

Consequently, the *aggR* gene mediates the expression of a large number of other EAEC genes responsible for virulence. The first genes found to be regulated by *aggR* were those encoding the aggregative adherence fimbriae (AAF)^11^*.* Our study findings demonstrate that an overall high incidence of the genomic strain of *aggR* was found across all the sites and the linear growth of children was negatively associated with the presence of *aggR* gene. However, though the role of *aggR* of EAEC in child growth remains unknown.

Our study findings also illustrate that the concomitant presence of *aaiC* and *aatA* strain carrying genes of EAEC was negatively associated with childhood linear growth except Nepal and Tazania. Other studies suggested that the presence of different virulence genes from within and outside the plasmid AA is necessary for complete EAEC virulence. Such aforementioned finding is in line with the findings of another concurrent study where the diagnosis of EAEC without overt diarrhea is associated with length/height-for-age z score decrements and chronic inflammation in children from Brazil^29, 30^.

Our data shows presence of improved floors in the house has a protective effect against infection of EAEC by *aaiC* genomic strain. We found higher maternal education was protective for *aaiC* and *Aar* genomic strain of EAEC. Findings from several other studies conducted in the MAL-ED settings also found an association of these factors with EAEC and *Campylobacter* infection^31, 32^.

The presence of cattle in the household was associated with *aaiC, Aar,* and *aggR* strain carrying genes*.* Still, there is no evidence regarding the association of the presence of an animal in the household with EAEC infection. The transfer of *Giardia lamblia* genes, any *E. coli* virulence gene, and unique *E. coli* virulence genes from animal feces to the hands of the mothers upon animal handling were reported in a study conducted in rural Bangladesh. As a result, domestic animals played a significant role in the spread of enteric pathogens in households^33^.

Previous findings have reported that EAEC detection was associated with higher levels of MPO (a marker for intestinal inflammation), NEO (intestinal inflammation), and AAT (permeability) among all 8 sites of MAL-ED^32^. In our study, the overall concomitant presence of *aaiC* and *aatA* strain carrying genes were more strongly associated with increased EED score, implying a higher intestinal inflammation. The relevance of elevated intestinal inflammation associated with virulence-related genes associated with EAEC is not yet clear and there is no evidence that virulence-related genes associated with EAEC were associated with elevated intestinal inflammatory biomarkers and subsequently an increased EED score. Henceforth, our study is the first attempt undertaken towards the generation of evidence-based understanding of the contribution of the different virulent genetic strains of EAEC with regards to enteric inflammation and poor child growth in LMIC settings.

Despite some potentially promising nature of our study findings, there are several possible limitations associated with our analysis. As an observational cohort study, the causality of the associations between infection with various genomic strains of EAEC and both intestinal inflammation and linear growth cannot be proven but can be hypothesized based on several factors, including the appropriate adjustment of the models for possible confounders, the strength and consistency of the associations, and the biological plausibility. We were unable to establish a temporal relationship between infections and the outcomes, which would require structured longitudinal models.

In conclusion poor maternal education, lack of improved floor, and ownership of cattle in the household are possible risk factors for EAEC infections by different genomic strains and thereby leading to a compromised linear growth in childhood. The burden of strain carrying genes associated with EAEC was associated with increased enteric inflammation among children in the first 2 years of life. EAEC virulence related genomic strains of *aaiC, Aar, aatA,* and the concomitant presence of *aaiC* and *aatA* genomic strains had a stronger association with growth failure among children of Bangladesh, whereas the association with inflammation was strongest for *aggR*, strain carrying genes.

## Method

### Study design and participants

MAL-ED (Etiology, Risk Factors, and Interactions of Enteric Infections and Malnutrition and the Consequences for Child Health) was a birth cohort study performed across eight sites in South America, sub-Saharan Africa, and Asia. The MAL-ED study design and methodology have been described elsewhere^34^. Briefly, 1715 children were enrolled from November 2009 to February 2012 from the community within 17 days of birth at all eight sites, namely: Bangladesh, India, Nepal, Pakistan, South Africa, Tanzania, Brazil, and Peru. In our current analysis, data from all 1715 participants were available from enrolment soon after birth up to 24 months of age.

### Data collection

Anthropometric measurements were done at monthly intervals up to the age of 24 months using standard scales (Seca gmbh & co. kg., Hamburg, Germany). Length-for-age z score (LAZ), weight-for-age z score (WAZ), and weight-for-length z score (WLZ) were calculated through the use of the 2006 WHO standards for children^35^. Anthropometric measurements were performed monthly. Details of illness and child feeding practices were collected during twice-weekly household visits^36^. Additionally, household demographics, presence of siblings, maternal characteristics and other data on the child’s birth and anthropometry were obtained at enrollment^34^. Beginning at 6 months of age, socioeconomic data were collected every 6 months. The WAMI score (Water, sanitation, hygiene, Asset, Maternal education, and Income index, ranging from 0 to 1) is a socioeconomic status index that includes access to improved water and sanitation, eight selected assets, maternal education, and household income as a representative of the socioeconomic status of the households^37^. A better socioeconomic status is indicated by a higher WAMI score^38^. Improved water and sanitation were defined following World Health Organization guidelines^39^. Treatment of drinking water was defined as filtering, boiling, or adding bleach^40^.

### Collection of stool and blood samples

Non-diarrheal stool samples were collected monthly (at least 3 days before or after a diarrhea episode) from birth to age 2 years and venous/peripheral blood was collected at 7, 15, and 24 months of age^41^. Raw stool aliquots and blood samples were processed at all sites using harmonized protocols and kept at − 80 °C freezers before subsequent laboratory analyzes^42^.

In this study, plasma zinc was assessed as the measure of zinc status at the age of 7, 15, and 24 months. Plasma zinc concentration is a proxy marker and recommended for the assessment of population zinc status, especially for children in low-income countries^43^. Plasma alpha-1-acid glycoprotein (AGP) level was considered as a biomarker for systemic inflammation and was also assessed at 7, 15, and 24 months^44^.

### Assessment of enteropathogens by TaqMan array cards (TAC)

Total nucleic acid (both DNA and RNA) was extracted from the fecal samples using the QIAamp Fast DNA Stool Mini kit (Qiagen), following the manufacturer’s guidelines. Two external controls, namely: MS2 bacteriophage and Phocine herpesvirus (PhHV) were added to the samples for the confirmation of nucleic acid extraction and amplification efficiency^45^.

For the detection of enteropathogens, a quantitative polymerase chain reaction (qPCR) with the use of a customized TaqMan Array Card (TAC) involving compartmentalized probe-based real-time PCR assays was used for the detection of a possible 29 pathogens from each of the samples^41^. Ct (quantification cycle) value of 35 was set as a threshold for analysis, whereby a Ct > 35 was considered as negative, as mentioned elsewhere^45^. In MAL-ED study, they investigated the occurrence of putative virulence-related genes (VRG) of *EAEC*, namely: *aatA*, aggR, Aar, and *aaiC*. To diagnose EAEC, the genes *aatA* (dispersin transporter protein), and *aaiC* (secreted protein) were targeted by PCR. Primers specific for EAEC identification were *aaiC* and *aatA*. Samples were considered positive for EAEC if they could detect either one of the two diagnostic genes or both. Only EAEC positive samples were further analyzed by multiplex PCRs to identify 20 EAEC VRG^46^. Moreover, cases positive for concomitant presence of both *aaiC* and *aatA* genotypes were further analyzed for co-infection and the list of pathogens in this study, as described elsewhere^42^. In MAL-ED Brazil site they compared the combinations of EAEC VRGs from positive samples presenting both diagnostic genes (*aaiC* and *aatA*). Their choice was based on the fact that only the samples presenting both genes were statistically associated with malnourished children ^46^.

### Assessment of biomarkers of intestinal inflammation

Intestinal inflammation was evaluated by measuring the levels of the biomarkers: alpha-1-anti-trypsin (Biovendor, Chandler, NC), neopterin (GenWay Biotech, San Diego, CA), and myeloperoxidase (Alpco, Salem, NH) in the stool samples collected from the study participants at the 3, 6, 9, 15, and 24 months of age time points by quantitative ELISA, using manufacturer’s guidelines^34^.

### Statistical analysis

All statistical analyses were performed in STATA 15 (Stata Corporation, College Station, TX). Descriptive statistics such as proportion, mean and standard deviation (SD) for symmetric data, and median with inter‐quartile range (IQR) for asymmetric quantitative variables were used to summarize the data. Incidence rates were calculated using Poisson regression where outcome variables were the number of infections of EAEC (different strain carrying genes) and offset variables were a log of number of follow up. The factors associated with strain carrying genes of EAEC in the monthly stool samples were calculated using Poisson regression models. In the final multiple Poisson regression model, the following variables were considered for inclusion using stepwise forward selection: child sex, birth weight, duration of exclusive breastfeeding in months, enrollment weight for age z-score, length for age z score, maternal age in years, maternal education, mother having less than 3 living children, maternal BMI, routine treatment of drinking water, improved sanitation, household ownership of cattle/poultry, and less than 2 people live in per room. We excluded children from the Pakistan site for growth analysis, owing to bias noted in a subset of this cohort within the study period. Myeloperoxidase (MPO), neopterin (NEO), and alpha-1-antitrypsin (AAT) values were log‐transformed before the analysis. At each time point, the composite EED score ranging from 0 to 10 was calculated from the three fecal markers, as described in the previous literature by MAL-ED researchers^20, 47^. Categories were assigned values as 0 (low), 1 (medium), or 2 (high). The formula for the composite EED score is as follows^48^:

$${\text{EED score}}\, = \,{2}\, \times \,{\text{AAT category}}\, + \,{2}\, \times \,{\text{MPO category}}\, + \,{1}\, \times \,{\text{NEO category}}$$

Associations between strain carrying genes of EAEC and composite EED score was estimated using generalized estimating equations (GEE) to fit regression models after adjusting for sex, age, water/sanitation, assets, maternal BMI, and (WAMI) index; enrollment length-for-age and weight-for-age z score, maternal height; poultry/ cattle in house, serum zinc level, inflammatory biomarker AGP (alpha-1-acid glycoprotein), presence of co-pathogens (*Campylobacter*, LT-ETEC, ST-ETEC, *Shigella*/EIEC, and *Giardia*), seasonality, and site for overall estimate and age in the month as time variable^49^. To assess and compare the associations of strain carrying genes of EAEC infection burden on growth at 24 months of age, we used multivariable linear regression after adjusting for the site and the necessary covariates. To detect multicollinearity, the variance inflation factor (VIF) was calculated, and no variable producing a VIF value > 5 was found in the final model. We calculated the strength of association by estimating the coefficient and its 95% CI (confidence interval). A *p*-value of < 0.05 was considered statistically significant during the multivariable analysis.

### Ethical consideration

The study was approved by the ethical committees at each of the participating institutes across each of the eight study sites^34^. The study was approved by the Research Review Committee and the Ethical Review Committee, icddr,b (BGD); Committee for Ethics in Research, Universidade Federal do Ceara; National Ethical Research Committee, Health Ministry, Council of National Health (BRF); Institutional Review Board, Christian Medical College, Vellore; Health Ministry Screening Committee, Indian Council of Medical Research (INV); Institutional Review Board, Institute of Medicine, Tribhuvan University; Ethical Review Board, Nepal Health Research Council; Institutional Review Board, Walter Reed Army Institute of Research (NEB); Institutional Review Board, Johns Hopkins University; PRISMA Ethics Committee; Health Ministry, Loreto (PEL); Ethical Review Committee, Aga Khan University (PKN); Health, Safety and Research Ethics Committee, University of Venda; Department of Health and Social Development, Limpopo Provincial Government (SAV); Medical Research Coordinating Committee, National Institute for Medical Research; Chief Medical Officer, Ministry of Health and Social Welfare (TZH)^32^.Written informed consent was obtained from the parents or legal guardians of every child.

## Data Availability

A publicly available MAL-ED dataset was analyzed in this study. This data can be obtained from here: ClinEpiDB [https://clinepidb.org/ce/app/record/dataset/DS_841a9f5259].
